# HeartMath as an Integrative, Personal, Social, and Global Healthcare System

**DOI:** 10.3390/healthcare10020376

**Published:** 2022-02-15

**Authors:** Stephen D. Edwards, David J. Edwards, Richard Honeycutt

**Affiliations:** Department of Psychology, University of Zululand, Private Bag X1001, KwaDlangezwa 3886, South Africa; edwards.davidjohn@googlemail.com (D.J.E.); rhoneycutt@triad.twcbc.com (R.H.)

**Keywords:** healthcare, HeartMath, Global Coherence Initiative, coherence, interconnectedness

## Abstract

COVID-19 is a recent major event, adding to planet Earth’s contexts of chaos, crime, injustice, illness, and violence. The HeartMath system has produced research evidence for scientific interventions that alter contexts characterized by chaos and stress, promoting health, coherence, and interconnectedness. This study provides an updated overview of HeartMath as an interdisciplinary, scientific, coherent, integral heart-based healthcare system, operated locally through various initiatives and globally through the Global Coherence Initiative. The HeartMath approach integrates ancient and contemporary, indigenous and mainstream, popular and folk, Eastern, Western, and African forms of healing. The HeartMath interdisciplinary, personal, social, and global vision and mission have considerable theoretical and practical potential for promoting planetary health, education, and development.

## 1. Introduction

COVID-19 is only one recent example of a factor influencing planet Earth’s contexts of chaos, incoherence, illness, and violence. In addition, endemic struggles for survival and subsistence stresses consume human energy, distort consciousness, and exacerbate inhumanity and disorder. All of this occurs within a planet in desperate need of healing. The compound word healthcare combines the quintessentially human notion of care with that of healing, in all its meanings: making whole, transferring from illness to health, and various forms of illness prevention and health promotion. At quantum information level, healthcare translates into dynamic human, social, and environmental energy patterns [1,2]. At a global level, healthcare needs to represent all inhabitants of planet Earth in their considerable diversity [1,2].

The HeartMath system has revealed various coherent, interconnected energetic patterns within and between human persons and populations [2]. As a concept, HeartMath may be operationally defined as the math of heart rate variability [HRV], particularly, heart rhythm variability, which varies coherently in optimal health and becomes disordered under stress as well as in various disorders and forms of illness [3]. HRV is recognized as key to unlocking the profound significance and meaning of the Morse-code-like information patterns communicated by the heart in its interactions with other bodily systems, particularly the brain. Healthcare information transfer is optimally facilitated during states of psychophysiological, personal, social, and global coherence, associated with stable, regular, rhythmic heart rate activity. In simple terms, independent of fast or slow heart rate, optimal heart rate variability and coherent heart rhythms indicate health and performance [2,3].

The HeartMath system was specifically created and developed by Doc Childre, a stress researcher who gathered an expert, interdisciplinary, multi-professional research team to reduce stress through the study and development of heart intelligence [1,3]. The special vision and mission of the HeartMath system is to promote personal, social, and global coherence and health [3]. Pioneering research has revealed profound patterns of heart communication involving human, energetic, electromagnetic, neurochemical, biophysical, and hormonal information [2,4,5]. Other studies have developed practical methods and techniques for stress reduction, health promotion, and performance enhancement [6], as well as biofeedback technology for heart rate variability (HRV) coherence training [3,7,8,9].

An overview follows of HeartMath as a scientific, coherent, integral heart-based, healthcare system that postulates and promotes planetary interconnectedness [10,11,12,13]. This system, its model, and perspectives are in line with some of the phenomenological insights of the ancient sages, meditative and contemplative traditions, as well as with integral theory [14,15] in a creatively evolving cosmos [7,13,16,17].

### Goal of This Study

This article was specifically motivated by the theoretical considerations and considerable evidence-based studies available in the HeartMath research library as to the scientific foundations and effectiveness of the HeartMath Institute in promoting coherent personal, social, and global healthcare. It is intended as an updated overview for instructional purposes.

## 2. The Math behind Heart Rate/Rhythm Variability Patterns

**Figure 1:**
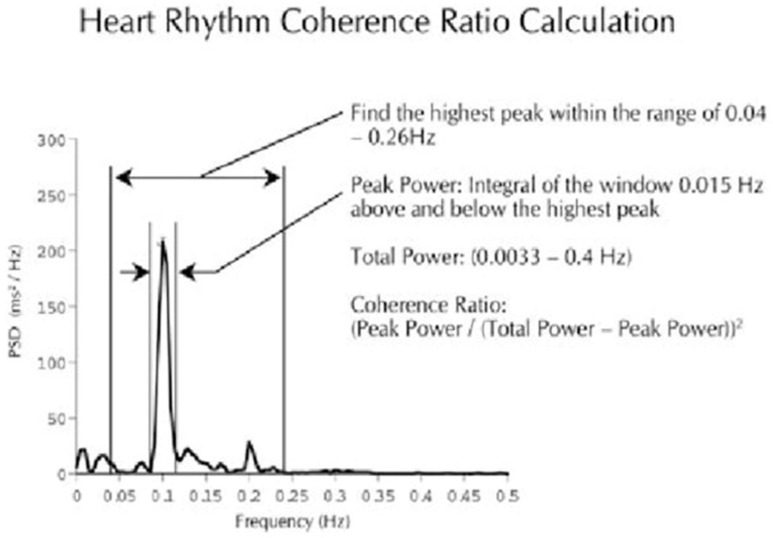
Indicates the HeartMath method of heart rhythm calculation [9]. The maximum peak is identified in the 0.04–0.26 Hz range in which coherence occurs. Peak power is determined by calculating the integral in a window that is 0.030 Hz wide. The total power of the entire spectrum is then calculated. The coherence ratio is formulated as: (Peak Power/(Total Power—Peak Power)) [2].

**Figure 2:**
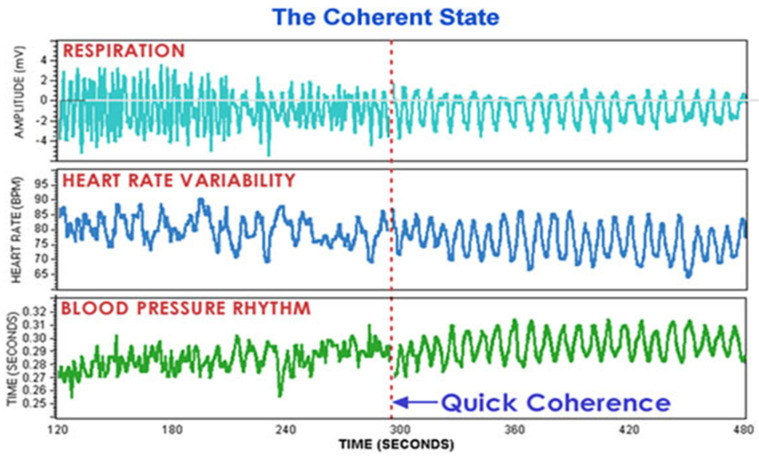
The Coherent State as reflected in synchronized entrainment of respiration, heart rate variability, and blood pressure rhythms brought about by the Quick Coherence technique.

Figure 1 graphically illustrates the HeartMath method of heart rhythm calculation. Figure 2 indicates synchronized entrainment, a healthcare intervention that can be brought about in minutes by the HeartMath Quick Coherence Technique [1,2,3,4,5,6,7,8,9].

## 3. The HeartMath System Is Unique in Its Recognition of the Integral Heart in All Its Physical, Emotional, Mental, Social, Ecological, and Spiritual Relatedness

From a transcultural perspective, the heart has received perennial recognition as the font of sentience, awareness, and consciousness; it is considered to be the center of the spiritual, intellectual, and emotional life. Various scientific disciplines provide evidence [18] that heart wisdom has developed over many millennia in central and southern Africa, predating the migrations of Homo sapiens through Asia and the subsequent development of the Vedanta, Yoga, and Chakra systems.

The integral heart appears to have dimensions of increasing depth: physical, emotional, transpersonal, and transcultural [3]. For example, in isiZulu, meanings of the heart include the physical organ, the seat of emotions, and the conscience [19]. Yoga views life-energy as flowing up and down the spine in three main pathways: the Ida, Pingala, and the Sushumna. These chakras are associated with particular anatomical locations of the nervous, endocrine, and other human functional systems [20,21]. The heart chakra expresses love and compassion [20,22]. Traditional Chinese medicine, especially Taoist chi-gung, is based on subtle consciousness/breath/energy work related to oscillating pacemaker cell rhythms that can be altered intentionally [21]. The Buddhist term for heart, sutra, is associated with ultimate enlightenment through the union of form and emptiness. Judaic energy centers (sefirot) include the harmony of the heart (tiferet). In the Kabbalah, the heart forms the central sphere [3]. In Christian Hesychastic and Sufi traditions, the Prayer of the Heart, which consists of the repetition of a phrase or the name of a deity, is intentionally accompanied by breath-paced heart focus. Centering prayer refers to a contemporary form of the Prayer of the Heart, which is very similar to the HeartMath Lock-In technique. In this context, Bourgeault [23] (p. 5) refers to the integral heart as a “homing magnetic center”, which is associated with a neurological shift in the mechanics of perception towards a unified field, where one becomes enabled to “see from wholeness”; this is discussed further in Section 7.

For millennia, the major great wisdom traditions, including Islam, Christianity, Buddhism, Hinduism, Taoism, and ancestral reverence [13,24,25], have espoused heart love. However, meaningful planetary effects have been slow, as the traditions have been engaged in continual disputes amongst themselves. Although many wisdom traditions advocate love, the practice of respect may be a more realistic goal for temporary transformation; Native American and many other indigenous traditions teach respect for all one’s relatives, namely, all of creation, as a primary duty. However, predictably endemic human destructiveness, greed, and power motivations will probably continue, especially since most people are still struggling to survive and satisfy their basic needs, let alone care for other, higher needs such as belongingness, love, and connection to greater Being [26]. 

Improved healthcare innovations concerning coherent, heartfelt intentions and actions are in short supply. 

## 4. HeartMath Healthcare Research

HeartMath is an interdisciplinary approach that bridges the natural, human, social, spiritual, and ecological sciences in its focus on heart-based research [9]. Coherence is a central concept. The fundamental vision and mission is to promote healthcare through research and education in personal, social, and global coherence.

Healthcare research findings from over 8000 researchers are freely downloadable from the research library 8000 compiled by HeartMath researchers. Over 400 additional studies undertaken by independent researchers have provided extensive external validity for the effectiveness of the HeartMath system, its methods, tools, and techniques. This is to be expected, as findings are generally based on evidence related to: 1. Physics; 2. Electromagnetics; 3. Cardiorespiratory activity, especially related to communication networks involving the vagus nerve; 4. The natural mechanism of respiratory sinus arrhythmia (RSA), whereby heart rate increases during inhalation and decreases during exhalation; 5. Coherent and incoherent heart rhythms, associated with positive and negative emotion, respectively; 6. Correlated heart rhythm mathematics; 7. Biofeedback conditioning principles and practices.

Pribram’s [17] holonomic, dynamic systems theory provides the theoretical, scientific model for much of the HeartMath research. For example, the model proposes complex pattern identification brain functioning, with special reference to emotion, the amygdala, and the cardiovascular system, especially the vagus nerve. In the model, experiential imprints stored as sets of familiar patterns in the neural architecture are continually monitoring and interacting with internal environmental inputs from many rhythmic physiological processes, such as heartbeat, respiration, and digestion, as well as with external environmental and social processes that help organize sensory perception, cognition, feelings, and behavior. According to the model, emotions are energetic happenings generated immediately from the occurrence of discontinuities, or novel patterns that do not match familiar, ongoing, and recurring inputs. Emotions, especially negative emotions, are known to highjack cognition via stress-induced, amygdala-generated fight/flight/freeze responses [3]. It is therefore postulated that a direct effect of utilizing HeartMath tools and techniques is the intentional facilitation of a heart-based re-patterning effect from physiological coherence [9]. It is also hypothesized that this operates at the physiological, emotional, and cognitive levels through afferent cardiac signals, a positive feeling pattern match, and the associated cortical electrophysiological activity. Rigorous research has provided empirical support for these hypotheses. After appropriate practice, their great value is the ability to transform the energy of negative emotions into their polar opposites, e.g., anger into assertiveness, overwhelming panic into centered motivation, overexcitement into relaxed release, sadness into contentment; hatred into love [2]. 

## 5. HeartMath Coherence Model

Theoretically, the HeartMath coherence model includes all the usual meanings of the term coherence, as implied in such terms as relationship, harmony, order, stability, consistency, synchrony, logic, and by the idea of the whole being more than the sum of its parts [8]. In academia, coherence refers to the internal integrity of an argument or thesis. Linguistically, it refers to intelligibility. Physiologically, the concept of coherence includes the synchrony of the circulatory and respiratory rhythms associated with overlapping sine wave patterns. In physics, it implies phase relationships. Auto-coherence, or auto-correlation, indicates stability in a single wave form; cross-coherence, among multiple waveforms, while phase locking and resonance include the concept of harmony in various rhythmic activities. In math and statistics, the term coherence implies correlation. In dynamic systems theory, it means connectedness, alignment, resonance, and optimal energy utilization.

From a psychophysiological perspective, coherence interconnects positive emotions with the cardiovascular, respiratory, immune, and nervous systems [9]. From a human as well as a social perspective, coherence applies to couples, teams, groups, organizations, and communities. Coherent relationships promote communication, synchronized behavior in rowing teams, and groups with similar goals. From a global perspective, communities and countries working cooperatively can cause ecological and planetary peace and harmony. Experientially, HeartMath praxis is accompanied by the sentient, increasing awareness of the synchronization of pulsation, respiration, and the renewing of positive feelings, whereby emotions such as peace and love are cumulatively experienced as radiating throughout the body, and among people and the wider world in harmonious interconnectedness.

Various HRV-related psychophysiological theories resonate with the HeartMath coherence model. Resonance theory is founded on heart rate variability biofeedback (HRVB) studies, which indicate that optimal heart rate oscillations occur via paced respiration at a frequency of about 0.1 Hz. Polyvagal theory, which hypothesizes social evolutionary mechanisms, advocates RSA and enhanced HRV for improved health and well-being. The neurovisceral integration model postulates a central autonomic network (CAN) related to social, cognitive, affective, and physiological regulation [9].

### 5.1. Psychophysiological Coherence

Psychophysiological studies indicate that bidirectional heart–brain communication has been recognized for over a century [27]. The heart possesses an intrinsic nervous system, capable of autonomous, functional decisions [9]. The heart communicates more with the brain than with any other organ [9]. Intricate heart rate variability patterns provide vital communicative links within the body, as well as between and among people, the ecology, and the cosmos. The sympathetic and parasympathetic (vagal) branches of the autonomic nervous system (ANS) function similarly to an accelerator and a brake, reflecting dynamic, resonant HRV patterns, signaling adaptation, resilience, and general health for diverse forms of healthcare assessment and intervention. HRV oscillations are typically categorized into very low frequency (VLF) bands between 0.0033 and 0.04 Hz, low frequency (LF) bands between 0.04 and 0.15 Hz, and high frequency (HF) bands from 0.15 to 0.4 Hz [28]. The psychophysiological coherence experience of zoned performance is closely related to Antonovsky’s [29] sense of coherence construct of the world as meaningful, manageable, and comprehensible, which also provides healthcare initiatives with a valid, unifying, theoretical, and practical rationale [30].

### 5.2. Social Coherence

Social coherence refers to harmonious relationships that facilitate efficient energy communication, cohesion, and action [8]. Effective multi-professional team functioning is ideal in many healthcare contexts. When nursing personnel, physiotherapists, medical practitioners, clinical psychologists, social workers, and occupational therapists effectively pool their particular areas of expertise in diagnostic and therapeutic contexts, patients heal. Hospitals implementing HeartMath programs have seen increased personal, team, and organizational functioning, as well as significant decreases in anxiety, depression, and anger. Studies provide evidence of functioning bioenergetics communication systems in highly coherent group contexts. Individuals with high heart coherence readily facilitate group coherence [8,31]. These findings have important implications for interpersonal group, family, and community psychotherapy. Studies indicate that emotional self-regulation skills and heart rhythm coherence training are associated with significant improvements in communication, employee satisfaction, productivity, problem solving, and significant returns on financial and social investments [8,31,32]. Numerous studies show that HRV coherence feedback facilitates self-regulation techniques and a wide range of health and performance outcomes.

### 5.3. Global Coherence

Ample evidence indicates that the Earth’s magnetic field generates and facilitates an interconnecting global information network [8,33,34]. Global Coherence Initiative (GCI) interconnectedness research from the Institute of HeartMath has established that there is a global network of magnetic field detectors around the planet, yielding information on human, planetary, and cosmic relationships. The GCI and the Global Consciousness Project (GCP) [34,35] provide a field view of human interconnectedness. Other related, emerging interdisciplinary field trends include neuroscience and cosmology [36]; the psychology of global consciousness [37] and co-created embodied spirituality [38]. The study of Timofejeva et al. [39] found synchronization between local magnetic field data, HRV wave rhythms, and interpersonal relationships. An integral Heart Based Resonant Frequencies [HBRF] theory of consciousness was postulated [40]. These findings support and extend those of many older studies concerned with transformations of consciousness, as particularly evident in moral behavior, creativity, and health promotion [41,42,43]. Conceptual and practical implications of this initiative, with special reference to global healthcare, are available from the websites: www.Heartmath.org and www.glcoherence.org (accessed on 4 April 2021). These websites also contain information on various online courses, for example, courses for any individual needing HeartMath Coach/Mentor training.

In a similar initiative, based upon his work researching the measurable effects of human intention [Steps for Moving Psychoenergetics Science Research Into the Hands of Interested General Public Researchers (filesusr.com accessed on 4 April 2021) and Steps for Moving Psychoenergetics Science Research Into the Hands of Interested General Public Researchers (filesusr.com accessed on 4 April 2021)], Stanford University Emeritus Professor William Tiller has initiated the Global Intention project through which concerned individuals the world over can create focused intention to improve our world. An example of this is the use of the intention suggested on the Global Intention|The Tiller Foundation website for healing the world from the effects of the COVID-19 virus. While in a meditative (coherent) state, participants create a positive intention that is “broadcast” worldwide.

## 6. HeartMath’s Healthcare Practice

HeartMath techniques and tools promote practical energetics, transform stress, strengthen resilience, and improve health. They typically emphasize heartfelt breathing in a ten-second cardio-respiratory rhythm, which facilitates RSA, rhythmic pulsation, and a focus on positive, renewing feelings [6]. Some traditional techniques include:

***Depletion to Renewal Grid*** The visualization of an energy graph of the hormonal system along the horizontal axis and the autonomic nervous system along the vertical axis facilitates the limiting of the negative effects of stress hormones such as cortisol and the increase of healthy hormones such as DHEA.

***Heart Focused Breathing*** is an effective, very brief meditation technique for slowing down fight, flight, and freeze reactions in order to focus on positive and renewing emotions, such as appreciation, peace, or love.

***Prep-Shift-Reset*** assists in resetting the energy system and building resilience for the rest of the day.

***Freeze-Frame*** consists in recognizing and “freeze-framing” any stressful feeling as if it were one static movie image, then practicing heart focused breathing, recalling a positive feeling, and finding a deep heart answer.

***Heart Lock-In*** facilitates deeper levels of heart experience. This technique is similar to other heart-based meditation, prayer and contemplation methods such as the Prayer of the Heart and the Arka Dhyana Intuitive Meditation, which will receive further discussion shortly.

***Cut-Thru*** addresses negative emotions triggered by situations, thoughts, and actions, and is typically associated with treating depression, anxiety, or anger [3].

***Coherent Communication*** improves relationships by cultivating personal coherence, clearly apprehending the other’s communication, and confirming the essence of that communication. When practiced regularly, coherent communication immediately increases empathy and Ubuntu and I-Thou relationships [44].

***Additional HeartMath (2020) tools*** are freely available at: https://www.heartmath.org/resources/downloads/12-heartmath-tools/, accessed on 4 April 2021.

Biofeedback instruments that have been scientifically developed to provide heart rate variability and heart rhythm coherence include:

***The emWave2***, which gives readings of and feedback on heart rate, heart rate variability, and physiological coherence. The instrument can be handheld for field use.

***The emWave Pro*** is a sophisticated coherence biofeedback program for use by professional specialists in education, psychology, medicine, etc.

***The Inner Balance*** Application (app) for personal coherence training is available to iPhone or smartphone users who download HeartMath programs from the internet.

***The Global Coherence (GC) app*** records mean coherence levels at individual, group and global levels. The app is freely downloadable from the internet. A HeartMath Pulse sensor provides biofeedback.

## 7. The HeartMath Website

The HeartMath website can be found at https://www.hearthmath.org/, accessed on 4 April 2021. The website and annual report provide further practical healthcare information. For example, it lists programs, including Add Heart and Children’s Heart Smarts and the 100,000 Coherent Kids Initiative, active in 93 countries. Since its inception, the HeartMath Institute has facilitated links with other health, educational, and research institutions. These established linkages are combined with the interdisciplinary originality and operational autonomy to lead to ground-breaking research in interdisciplinary fields such as biofield physiology and vibroengineering, which have impressed with their robust, scientific grounding [36].

## 8. HeartMath Global Healthcare Meditation

As concerns in-depth personal healthcare, the Heart Lock-In is one of the earliest HeartMath techniques developed. In a recently published study, 104 participants from five countries completed 15 days of ambulatory HRV monitoring. Analysis of participants HRV before, during, and after a Heart Lock-In meditation period indicated significantly increased coherence, as well as correlation with magnetic field activity on the day of the meditation [45].

This study may be regarded as cutting-edge quantitative scientific evidence for the claims of many earlier mass meditation studies to decrease violence and facilitate moral consciousness and behaviour, creativity, and health promotion [46]. From a qualitative perspective, an in-depth coherence experience has been described as follows [9,47]:

“Beyond the unique nature of each event, with their individual integrity and superficial differences, the essential structure of the coherence experience initially appears as some variation on the conscious practise of the cardiorespiratory, rhythmic process of breath connecting heart beats, warming and softening the heart, heralding a sense of stillness, alignment, harmony and peace, as scattered energy is felt to collect in the heart area, bringing deepening heart awareness and, typically, at some point a sense of “lift off” to unlimited self or space. This gathering energy seems to be distributed throughout the body and beyond, resonating with increasing subtlety and/or refinement into a higher, vibrational level typically experienced as love, accompanied by finer feelings of centeredness, wholeness, oneness and interconnectedness. Unique, individual experiences vary. They may be concrete, abstract, diffuse, definite, ordinary, mixed, mystical and/or paradoxical, of, for example, homecoming, unboundedness, spaciousness, timelessness, emptiness, freedom, happiness, bliss, joy and infinite creativity. Experiences typically have local, social or global action implications, insights, intuitions and moral directions, as, for example, for “making the world a better place” through writing, healing and teaching”.

From this qualitative experiential perspective, it is instructive to note the great wisdom traditions of Judaism, Christianity, and Islam view the heart as the principal organ of spiritual perception. The great healing value of the Prayer of the Heart is commonly extolled by Christian and Sufi mystics [23,48]. HeartMath technology has also recently indicated highly significant increases in both coherence and achievement in the use of a similar heart-based method called Arka Dhyana, or Intuitive Meditation. Authors postulate that the HeartMath Coherence Model cast new light on the ancient Yogic idea of yoking in relation to embodied spirituality, both with regard to integration of the diverse bodily energies in immanent spirituality and holistic divine heart-based transcendence.

## 9. Conclusions

The HeartMath Institute formally reviews its activities in the form of annual reports. A variety of these activities regularly feature in update formal reviews by independent researchers, more of which are needed [49,50]. The specific goal of this study was to provide an inclusive, update, overview of HeartMath as an integrative, personal, social, and global healthcare system. Various research studies have consistently endorsed the practical value of diverse HeartMath tools, techniques, and electronics as excellent in transforming negativity into positivity and renewing the patterns of energy typically experienced in the forms of such feelings as peace and love. Considerable scientific evidence points to vast, energetic, interconnectivity at the human, planetary, and solar systemic levels. It is reasonable to conclude that the many HeartMath practitioners and GCI ambassadors from over 150 countries [2,3] who practice heart focused care, compassion, and love with an aim towards improving global coherence provide substantial human and planetary healthcare.

The present study has provided an overview of HeartMath as a coherent, integral heart-based healthcare system. Various forms of evidence have been presented to support this contention. There does not appear to be any contradictory evidence to this effect, although further gold-standard, empirically orientated, randomized controlled trials are needed [50]. Future conceptual, theoretical, and paradigmatic studies are also needed. In practical healthcare terms, positive emotions and heart focused breathing may facilitate vast interconnectivity. In addition to the pursuit of scientific programs and interventions, global healthcare amongst the general public needs vigorous promotion, especially when aimed towards facilitating the coherent collaboration of all related healthcare organizations and their associated stakeholders.

## Data Availability

Not applicable.
